# Unraveling the emerging role of glial heterogeneity in neuropathic pain: from pathological mechanisms to therapeutic Frontiers

**DOI:** 10.3389/fneur.2026.1820914

**Published:** 2026-05-28

**Authors:** Zhiwei Li, Jiafeng Lu, Zhonghua Chen

**Affiliations:** 1 School of Medicine, Shaoxing University, Shaoxing, Zhejiang, China; 2 Medical Research Center, Shaoxing People’s Hospital, Shaoxing, Zhejiang, China; 3 Department of Anesthesiology, Shaoxing People’s Hospital, Shaoxing, Zhejiang, China

**Keywords:** cell communication, glial cell heterogeneity, neuroinflammation, neuropathic pain, pain regulation

## Abstract

Neuropathic pain (NP) arises from injury or dysfunction within the somatosensory nervous system and represents a major clinical challenge due to its complex and multifactorial pathogenesis. Emerging evidence underscores that the onset and maintenance of NP are not solely governed by neuronal mechanisms but are critically shaped by the persistent activation and inherent heterogeneity of glial cells. Glial heterogeneity encompasses diverse molecular phenotypes, functional states, and distinct spatial distributions. Within the central nervous system (CNS), microglia and astrocytes undergo dynamic phenotypic transitions, contributing to both neurotoxic and neuroprotective effects by modulating neuroinflammatory cascades. In the peripheral nervous system, satellite glial cells actively sensitize sensory neurons through enhanced intercellular communication and the release of specific mediators, thereby facilitating the development and persistence of NP. The coordinated actions of heterogeneous glial populations drive key pathological processes—including synaptic remodeling, sustained neuroinflammation, and dysregulation of ion channels—ultimately promoting peripheral and central sensitization. Importantly, emerging therapeutic strategies targeting distinct glial subpopulations or their specific activation states, such as P2X4 receptor antagonists or NF-κB inhibitors, have shown promise beyond conventional neuron-centric approaches. This review synthesizes current insights into glial heterogeneity in NP, addressing a critical gap in the literature and providing a framework for advancing mechanism-based clinical interventions.

## 1 Introduction

Neuropathic pain (NP) is a chronic pain syndrome caused by lesion or disease of the somatosensory nervous system (1, 2). Its clinical hallmark is pain elicited by non-noxious stimuli, often accompanied by spontaneous episodes of severe pain (3, 4). Epidemiological data indicate that NP affects approximately 3–17% of the global population (5), with about 7–10% of patients exhibiting abnormal hyperalgesia (6). Based on the anatomical site of injury or lesion, NP is classified into central and peripheral types; peripheral NP is more prevalent clinically, with representative conditions including painful diabetic peripheral neuropathy, postherpetic neuralgia, and trigeminal neuralgia (7). Beyond pain, patients frequently experience paresthesias such as tingling and burning sensations. These symptoms contribute to motor dysfunction and psychological deterioration and impose a substantial health economic burden on individuals and society (8, 9).

With advances in neuroscientific techniques and a deeper understanding of the pathophysiological mechanisms underlying NP, the conceptual framework has shifted from a predominantly neuron-centric model to a glia-centric model of neural dysfunction. Glial cells, which constitute the major non-neuronal population of the central nervous system (CNS), include microglia, astrocytes, oligodendrocytes, and satellite glial cells (SGCs). Under homeostatic conditions, these cells support neurons, supply metabolic substrates, and preserve ionic and transmitter homeostasis (10). Compelling evidence now indicates that glial cells are not passive bystanders but active participants and key drivers in both the initiation and the maintenance of NP (11). This conceptual reorientation highlights that aberrant glial reactivity and maladaptive neuron–glial communication are central pathogenic events, rather than mere epiphenomena of neuronal injury.

**Figure 1:**
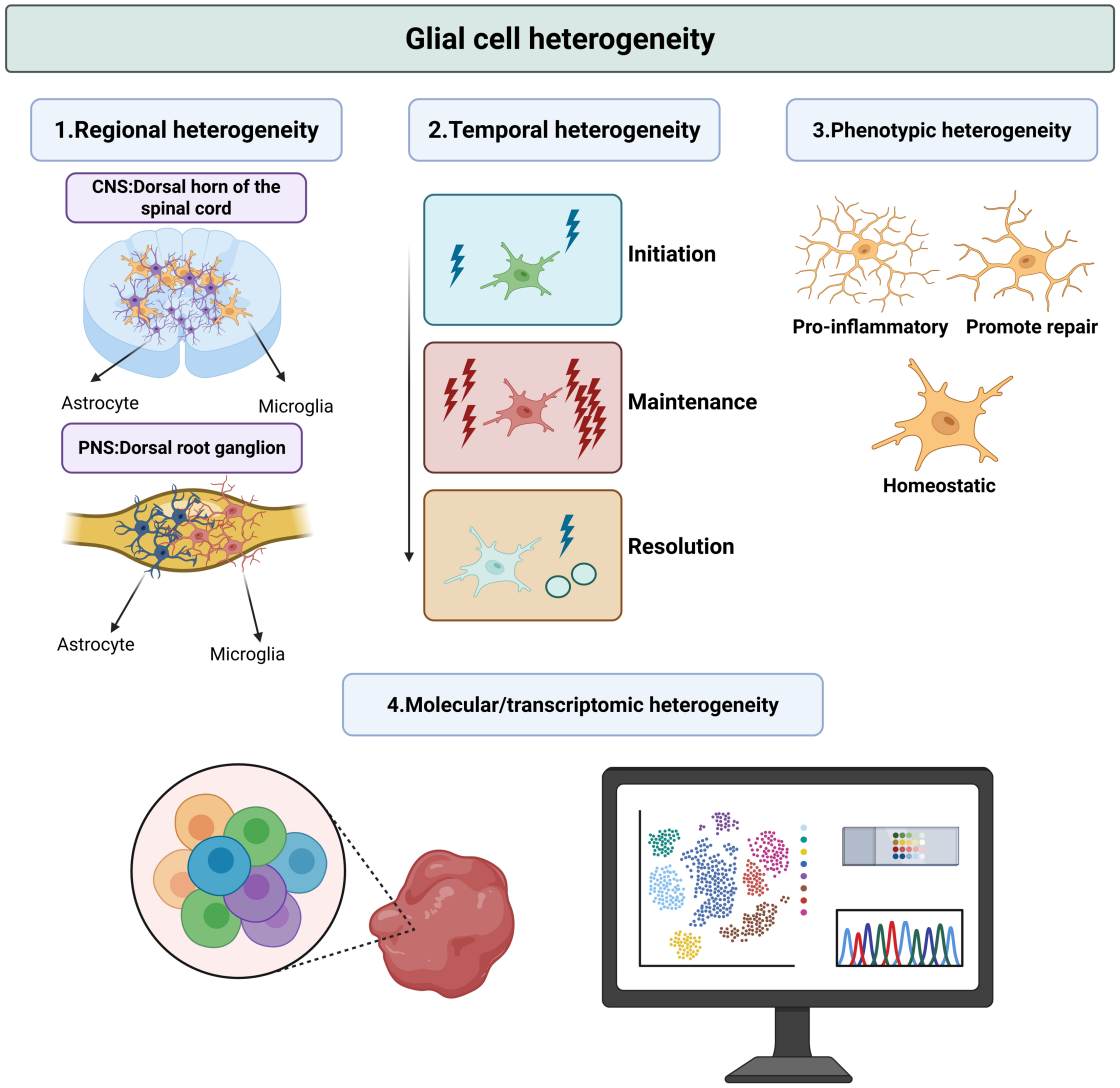
The concept of glial cell heterogeneity. This figure legend primarily introduces the concept of glial heterogeneity. Glial heterogeneity encompasses four key dimensions: spatial (regional differences), temporal (phase-specific roles during pain), phenotypic (polarization states and marker profiles), and molecular/transcriptomic (subpopulation diversity revealed by omics).

A central tenet of the glia-centric framework is the recognition of glial heterogeneity. Glial heterogeneity manifests across four principal dimensions: (i) spatial heterogeneity (regional and subregional variation, such as differences between the spinal dorsal horn and the dorsal root ganglion), (ii) temporal heterogeneity (differential contributions during the initiation, maintenance, and resolution phases of pain), (iii) phenotypic heterogeneity (divergence in functional polarization states and marker expression profiles), and (iv) molecular/transcriptomic heterogeneity (subpopulation diversity revealed by single-cell and spatial omics technologies) (12–14) (Figure 1). In the context of NP, distinct glial subtypes and activation states appear to be recruited in a stage- and region-specific manner. For instance, following peripheral nerve injury, spinal microglia undergo rapid morphological and transcriptional remodeling and are thought to play a critical role in the early initiation phase of pain (15), whereas astrocytic reactivity and network reorganization have been more closely associated with the chronic maintenance of pain hypersensitivity (16). Similarly, within peripheral ganglia, satellite glial cells exhibit spatial heterogeneity that may influence neuronal excitability through mechanisms including Kir4.1 channel modulation (17). Furthermore, oligodendrocyte lineage cells—traditionally investigated primarily in the context of axonal myelination—have been implicated in pain processing, although the extent to which their spatial and temporal dynamics contribute to NP remains incompletely defined and warrants further investigation (18). Elucidating such heterogeneity is essential for addressing current gaps in the mechanistic framework of NP and may help explain why distinct etiologies give rise to pain phenotypes that are both overlapping and etiologically distinct.

A systematic dissection of glial heterogeneity not only refines the pathological taxonomy of NP but also illuminates a spectrum of mechanism-based, subtype-specific therapeutic opportunities. Recent studies leveraging single-cell and spatial transcriptomic profiling of the DRG and spinal dorsal horn have clarified key molecular nodes in aberrant neuron–glial crosstalk (19), thereby nominating potential targets for precision intervention. However, several critical knowledge gaps persist: the precise role of SGC-specific mechanisms (e.g., Kir4.1-mediated regulation) in trigeminal pain pathways remains incompletely defined, the functional contribution of oligodendroglial heterogeneity to NP pathogenesis is only beginning to be explored, and the translational relevance of transcriptomically defined glial states awaits rigorous validation in human tissues. Therefore, a comprehensive synthesis of the relationship between glial cell heterogeneity and NP will provide an indispensable theoretical foundation for deepening our understanding of pain pathogenesis, for identifying novel therapeutic targets, and for devising more precise, stage-tailored treatment strategies.

## 2 Pathogenesis of NP

### 2.1 Classical pathogenesis of NP

The pathogenesis of NP involves multiple processes, including peripheral sensitization, central sensitization, and dysfunction of descending inhibitory systems (20). Central sensitization is considered the core mechanism sustaining pain (21). Peripheral sensitization refers to an abnormal increase in the sensitivity of peripheral nociceptive neurons to afferent signals. After peripheral nerve injury, cells at the injury site and infiltrating inflammatory cells (e.g., mast cells, lymphocytes) release chemical mediators such as norepinephrine, bradykinin, histamine, and prostaglandins, leading to nociceptor sensitization and amplification of pain signal transmission. Central sensitization manifests as heightened excitability and enhanced synaptic transmission of pain-related neurons at spinal and supraspinal levels, resulting in pathological changes including increased neuronal spontaneous discharge, expanded receptive fields, reduced stimulus thresholds, and enhanced responses to supra-threshold stimuli. Corresponding clinical manifestations include spontaneous pain, hyperalgesia, and allodynia (22–24). Additionally, dysfunction of descending inhibitory systems is mainly associated with enhanced activity of *μ*-opioid receptor (MOR) neurons in the rostral ventromedial medulla, producing descending facilitatory effects and weakening normal descending inhibition (25).

### 2.2 A common role in the pathogenesis of NP

During the progression of these mechanisms, glial heterogeneity represents a prominent pathological feature of NP and contributes to amplification and maintenance of pain signals through multiple pathways (26, 27). At different stages of pain development, phenotypic shifts and functional diversity of microglia and astrocytes plays a key role (28). Interaction between glial cells and neurons is a critical mechanism underlying pain signal amplification. Distinct astrocyte subsets enhance pain transmission through neuron-glial and glial-glial communication networks (29). In this context, connexins, which are transmembrane proteins mediating intercellular communication, play an important role (30). Moreover, excessive release of *γ*-aminobutyric acid (GABA) by reactive astrocytes in the spinal cord contributes to regulation of pain signal conduction (31). Heterogeneous glial cells further modulate neuronal excitability by releasing mediators, including pro-inflammatory cytokines, ATP, and matrix metalloproteinases (32). Among these, aberrant MMP activation contributes to NP development through multiple mechanisms, including disruption of myelin structure in peripheral nerves and the spinal dorsal horn, increased excitability of DRG neurons, activation of NMDA receptors, promotion of heterogeneous glial responses, and facilitation of pro-inflammatory cytokine release (33).

## 3 Glial cell heterogeneity in NP

In the nervous system, glial cells exhibit pronounced heterogeneity, a characteristic that profoundly influences their functional properties under both physiological and pathological conditions. In NP, glial heterogeneity contributes to pain modulation through multiple mechanisms, primarily manifested as differential effects on synaptic remodeling, neural circuit function, and inflammatory signaling pathways (34).

### 3.1 Involvement of glial cells in pain processing via modulation of synaptic plasticity and neural circuits

In the spinal dorsal horn, activated microglia contribute to central sensitization by modulating functions such as GABAergic inhibitory synapses. For example, Spinal pro-inflammatory microglia suppress GABAergic signaling via activation of the ATP-P2X4R signaling pathway, thereby promoting the transition from acute pain to chronic pain remodeling (35). Furthermore, pro-inflammatory microglia enhance excitatory glutamatergic transmission and attenuate inhibitory GABAergic and glycinergic transmission in the spinal dorsal horn through the release of tertiary mediators, including BDNF, TNF-*α*, and IL-1β, thereby facilitating the generation and propagation of nociceptive information and ultimately inducing central sensitization that sustains persistent pain (36). Interestingly, animal studies have demonstrated that reactive astrocytes reverse inhibitory GABAergic signaling into excitatory signaling through excessive GABA release and alterations in cellular electrogenic properties, thereby leading to central sensitization (31).

### 3.2 Modulation of pain by glial cells through subtype-specific neuroinflammation

ATP activates microglia through P2X4R and P2X7R receptors, inducing the release of BDNF and IL-1β and enhancing neuronal excitability (33). The CX3CL1-CX3CR1 chemokine axis further strengthens microglia–neuron interactions, thereby aggravating neuroinflammation (31). In astrocytes, activation of the IL-6/JAK/STAT3 pathway drives pro-inflammatory polarization (e.g., A1 type) and neuroinflammatory responses, and inhibition of STAT3 by Bt354 significantly alleviates pain behaviors (37). Galectin-3 (Gal3) released from injured sensory neurons also activates spinal microglia and potentiates excitatory synaptic transmission (38). Glial-derived inflammatory mediators such as TNF-*α* increase neuronal firing by modulating ion channels, including Nav1.3 and Cav3.2, resulting in hyperalgesia (39). Concurrent downregulation of Kv4.2 potassium channels reduces intrinsic neuronal inhibition and further amplifies pain signaling (40). Concurrently, astrocytes regulate extracellular glutamate levels by modulating glutamate transporter function, including GLT-1, thereby altering synaptic plasticity and pain sensitivity (29).

In summary, glial cells represent a highly heterogeneous population whose molecular and cellular diversity governs their contribution to NP. Through coordinated effects on synaptic remodeling, circuit dysfunction, and inflammatory signaling, heterogeneous glial responses collectively drive NP onset and progression. Elucidation of these mechanisms provides a critical theoretical foundation for targeted and precise pain intervention.

### 3.3 Microglial heterogeneity and NP

#### 3.3.1 Regional heterogeneity of microglia under physiological conditions

**Figure 2:**
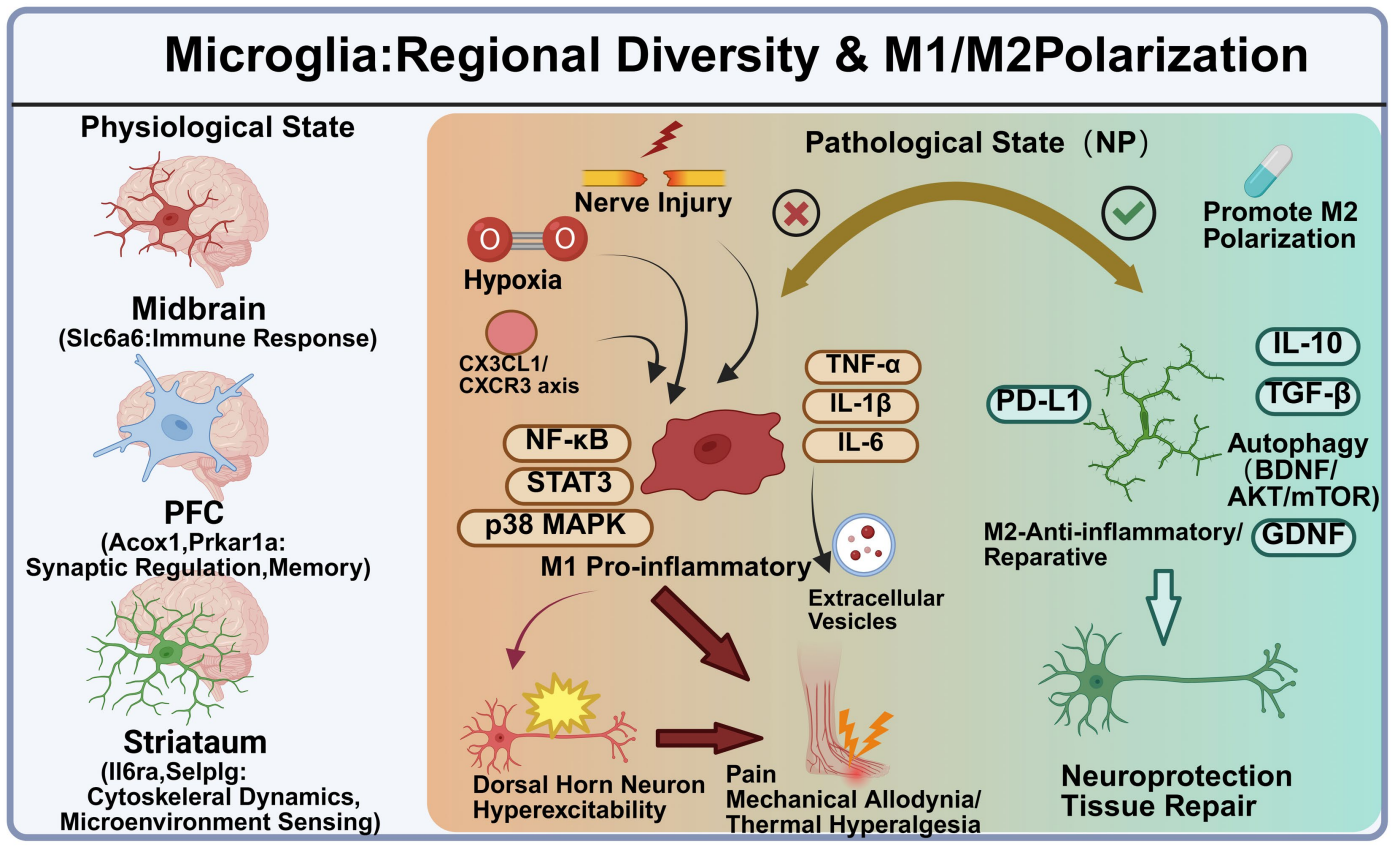
Physiological manifestations of microglial heterogeneity and its pathological alterations in NP. This figure summarizes key roles of microglia in NP. Microglia: As resident immune cells of the central nervous system, they exhibit significant region-specific functional heterogeneity. In NP, microglia are activated and undergo dynamic phenotypic polarization, primarily into the pro-inflammatory M1 state and the anti-inflammatory/reparative M2 state. The M1 phenotype promotes neuronal hyperexcitability in the dorsal horn and exacerbates pain by releasing mediators such as TNF-*α* and IL-1β, whereas the M2 phenotype exerts neuroprotective and anti-inflammatory effects via secretion of IL-10 and neurotrophic factors. The dynamic M1/M2 balance, regulated by microenvironmental signals, is crucial for NP progression.

Microglia are resident immune cells of the CNS that perform essential defensive functions, including phagocytosis of apoptotic debris and abnormal proteins, while also regulating neural circuits and maintaining homeostasis through continuous microenvironmental surveillance and secretion of regulatory factors. Under physiological conditions, microglia exhibit significant heterogeneity, reflected in region-specific transcriptomic profiles and functional specializations. For instance, midbrain microglia preferentially express immune- and interferon-related genes associated with antigen presentation (e.g., Slc6a6), whereas prefrontal cortical microglia are enriched in genes involved in synaptic regulation and long-term memory processes (e.g., Acox1, Prkar1a). In contrast, striatal microglia show elevated expression of genes such as Il6ra and Selplg, associated with cytoskeletal organization and microenvironmental sensing (41). These transcriptomic findings are derived from preclinical rodent studies and have not yet been validated in human postmortem brain tissue at comparable resolution. This regional specialization provides a cellular basis for the diverse contributions of microglia to NP pathophysiology (Figure 2; Table 1).

**Table 1:** Heterogeneity of microglia and astrocytes and their roles in neuropathic pain.

Cell type	Polarization state	Markers	Functional characteristics	References
Microglia	Physiological state–Midbrain	Slc6a6	Express immune–and interferon-related genes associated with antigen presentation	(41)
Microglia	Physiological state–Prefrontal cortex	Acox1, Prkar1a	Involved in synaptic regulation and long-term memory processes	(41)
Microglia	Physiological state–Striatum	Il6ra, Selplg	Cytoskeletal organization and microenvironmental sensing	(41)
Microglia	M1 (pro-inflammatory)	CD86, iNOS	Secrete TNF-α, IL-1β, IL-6; promote dorsal horn neuronal hyperexcitability, drive mechanical allodynia and thermal hyperalgesia; release extracellular vesicles that amplify pain signaling	(42–46)
Microglia	M2 (anti-inflammatory/reparative)	Arg-1, CD206, Ym1/2	Anti-inflammatory, neuroprotective; secrete IL-10, TGF-β, BDNF, GDNF	(47, 48)
Microglia	Dynamic regulation (M1/M2 balance)		Regulated by cytokine microenvironment, metabolic state (choline transporter function), etc.	(49, 50)
Astrocytes	Physiological state–Hippocampus	Ferritin	Regulate local iron homeostasis	(51–53)
Astrocytes	Physiological state–Cortex	Ang-1, TGF-β	Maintain blood–brain barrier integrity	(51–53)
Astrocytes	Physiological state–Cerebellum	GAT-1	Facilitate rapid GABA clearance and motor learning	(51–53)
Astrocytes	Physiological state– Gray vs. white matter	Not specified	Gray matter: regulate synaptic transmission and pain signaling; white matter: support metabolic homeostasis and ionic balance	(54–56)
Astrocytes	A1 (pro-inflammatory/neurotoxic)	C3, SAA3	Release IL-1β, TNF-α; suppress GLT-1/GLAST leading to glutamate excitotoxicity; secrete MCP-1/CCL2 recruiting immune cells; sustain phenotype via C3-C3aR positive feedback loop	(57–62)
Astrocytes	A2 (anti-inflammatory/neuroprotective)	S100a10, Tgm1	Secrete BDNF, GDNF, NGF; upregulate EAAT2 to enhance glutamate clearance; release IL-10, TGF-β; support extracellular matrix remodeling and tissue repair	(57, 63, 64)
Astrocytes	Dynamic regulation (A1/A2 balance)		Regulated by NLRP3 inflammasome, Lcn2, PK2, etc.	(65, 66)

#### 3.3.2 The M1/M2 polarization framework in NP: utility and limitations

During NP progression, microglia undergo activation and exhibit dynamic phenotypic polarization, which has been heuristically categorized—primarily based on *in vitro* stimulation paradigms and surface marker expression—into M1 (pro-inflammatory) and M2 (anti-inflammatory or reparative) states. It is critical to acknowledge that the binary M1/M2 classification represents a simplified, operational model rather than a definitive reflection of the *in vivo* activation landscape mapped by single-cell transcriptomics or epigenomic profiling. Indeed, recent genetic evidence indicates that activated microglia in the injured CNS frequently occupy intermediate or hybrid transcriptional states that are not fully captured by canonical M1/M2 markers, and the majority of supporting data for this polarization model originate from *in vitro* cell culture systems and preclinical animal models, with limited direct validation in human NP conditions.

M1 polarization is induced by pathological signals including CX3CL1/CX3CR1 signaling and hypoxic stress and is mediated through activation of STAT3, NF-κB, and p38 MAPK pathways. M1 microglia upregulate markers such as CD86 and iNOS and secrete high levels of pro-inflammatory mediators, including TNF-*α*, IL-1β, and IL-6, which promote dorsal horn neuronal hyperexcitability and drive mechanical allodynia and thermal hyperalgesia. Preclinical evidence from experimental models such as chronic constriction injury and spinal cord injury demonstrates that pharmacological suppression of M1 polarization using agents such as procaine, paeonol, or lidocaine significantly attenuates pain behaviors (42–45). Additionally, extracellular vesicles and related mediators released by M1 microglia have been shown in animal studies to contribute to the amplification and propagation of pain signaling (46) (Figure 2) (Table 1).

In contrast, M2 microglia express markers such as Arg-1, CD206, and Ym1/2 and exert anti-inflammatory, neuroprotective, and functions through secretion of anti-inflammatory mediators including IL-10 and TGF-*β*, as well as brain- and glial cell-derive neurotrophic factors (BDNF and GDNF). In preclinical NP models, enhancement of M2 polarization—for example, through electroacupuncture—has been associated with significant improvement in pain symptoms. Underlying regulatory mechanisms reported from experimental studies involve molecular pathways including PD-L1 signaling, autophagy-related BDNF/AKT/mTOR signaling, and NF-κB modulation (47, 48) (Figure 2) (Table 1). Notably, while these findings underscore the potential therapeutic relevance of shifting microglial phenotypes, direct clinical evidence linking M2-polarized microglia to pain relief in patients remains sparse.

#### 3.3.3 Dynamic regulation of microglial phenotypic state

Accumulating evidence indicates that microglial phenotypic polarization is not static; rather, the M1/M2 balance is dynamically regulated by factors such as the cytokine microenvironment, cellular metabolic state, including choline transporter function, and signaling pathways such as GDF11/p38 MAPK (49, 50) (Table 1). Given the aforementioned limitations of the binary classification, emerging research increasingly emphasizes a continuum of activation states that are shaped by the specific pathological milieu, rather than two discrete cell fates. This dynamic balance critically influences NP progression and outcome in experimental models.

#### 3.3.4 Concluding remarks and therapeutic implications

In conclusion, microglia regulate NP through their intrinsic regional heterogeneity and phenotypic plasticity, with the heuristic M1/M2 framework providing a useful conceptual scaffold for understanding the pro- and anti-inflammatory poles of microglial activity. Nevertheless, future studies must incorporate more nuanced, transcriptomically defined activation signatures to fully delineate the role of microglial states in human NP. Therapeutic strategies aimed at modulating the overall microglial functional balance hold promise but require further validation in clinical cohorts, given that current evidence is derived almost exclusively from preclinical investigations.

### 3.4 Astrocytic heterogeneity and NP

#### 3.4.1 Physiological heterogeneity and homeostatic functions of astrocytes

**Figure 3:**
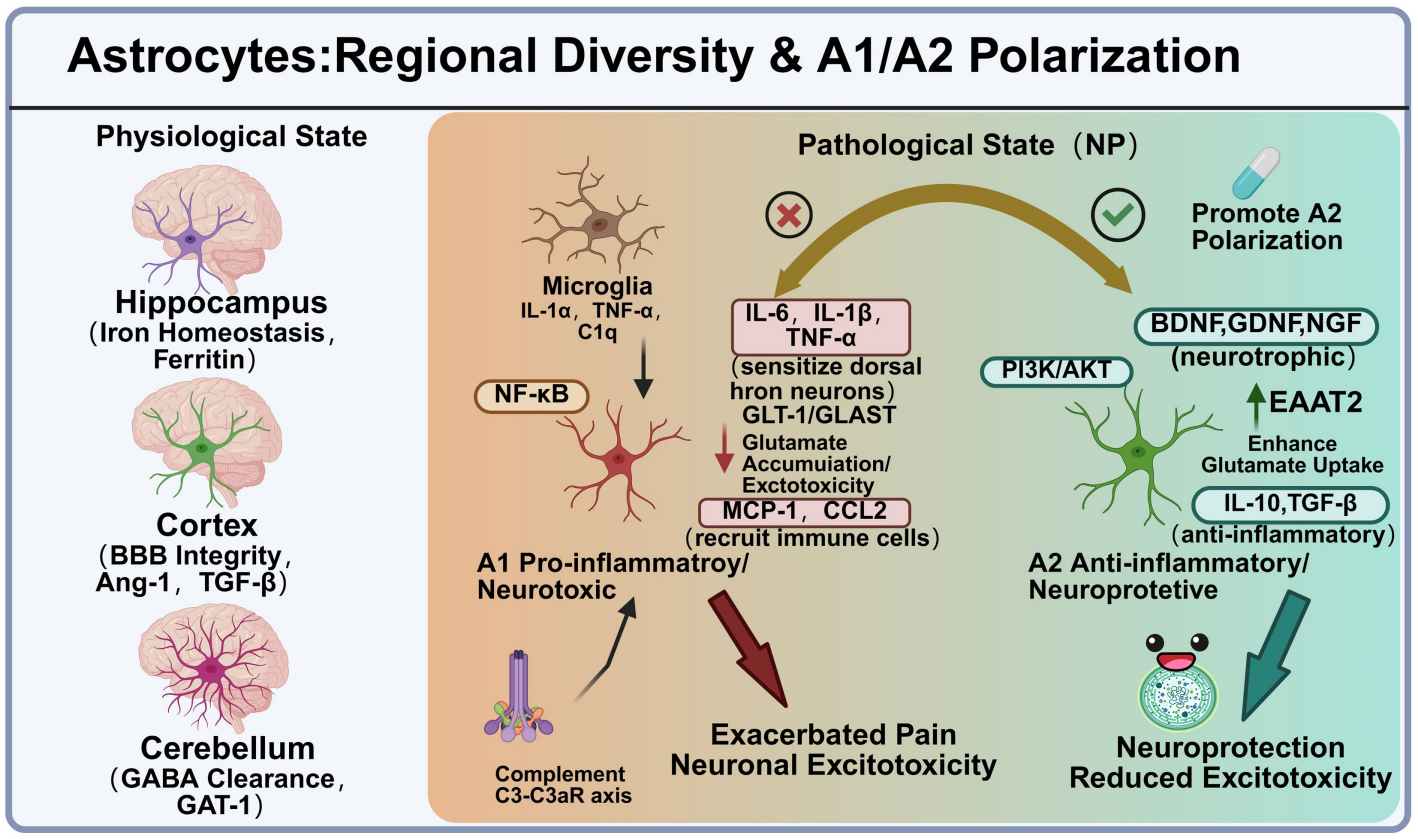
Physiological manifestations of astrocyte heterogeneity and its pathological changes in NP. This figure summarizes key roles of astrocytes in NP. Astrocytes: These are the most abundant and functionally diverse glial cells. In NP, they polarize into the neurotoxic A1 phenotype and the neuroprotective A2 phenotype. The A1 phenotype, induced by factors released from microglia, aggravates pain and neuroinflammation by releasing inflammatory cytokines and reducing glutamate uptake. The A2 phenotype promotes repair by enhancing glutamate clearance and secreting anti-inflammatory factors and neurotrophic factors. Restoring the A1/A2 balance is a potential therapeutic strategy.

Astrocytes are the most abundant and functionally diverse glial cells in the CNS. They contribute to blood–brain barrier formation through endfoot contact with blood vessels and regulate cerebral microcirculation and metabolic homeostasis. Simultaneously, by ensheathing synapses, recycling neurotransmitters, and releasing neurotrophic factors, astrocytes provide essential structural and functional support for neural circuit development and maintenance. Astrocytic functions display marked regional heterogeneity. For example, hippocampal astrocytes regulate local iron homeostasis through ferritin enrichment, cortical astrocytes preserve blood–brain barrier integrity by secreting Ang-1 and TGF-*β*, and cerebellar astrocytes express high levels of GAT-1 to facilitate rapid GABA clearance and motor learning (51–53) (Figure 3). In NP, preclinical studies have shown that astrocytes in gray and white matter exhibit functional divergence, with gray matter astrocytes preferentially regulating synaptic transmission and pain signaling, whereas white matter astrocytes primarily support metabolic homeostasis and ionic balance (54–56). The extent to which these region-specific functional specializations are conserved in the human spinal cord and brain under chronic pain conditions remains an active area of investigation (Table 1).

#### 3.4.2 The A1/A2 polarization framework in NP: heuristic value and conceptual constraints

Under pathological conditions, the complex heterogeneity of reactive astrocytes has been widely conceptualized through a simplified, operational framework— largely derived from transcriptomic responses to defined *in vitro* stimuli—that categorizes these cells into A1 (pro-inflammatory and neurotoxic) and A2 (anti-inflammatory and neuroprotective) phenotypes. It is essential to underscore that the binary A1/A2 classification represents an investigative tool and a conceptual simplification rather than a precise genomic or epigenomic map of astrocytic activation states *in vivo*. Emerging evidence from single-nucleus RNA sequencing and spatial transcriptomics reveals that reactive astrocytes in the injured or diseased CNS adopt a wide spectrum of context-dependent transcriptional states that frequently do not align with the canonical A1 or A2 profiles. Furthermore, the overwhelming majority of data supporting the A1/A2 model derives from *in vitro* astrocyte cultures and preclinical animal models; direct clinical evidence linking specific A1- or A2-like signatures to pain outcomes in human patients remains extremely limited.

A1 astrocytes have been described as exhibiting pro-inflammatory and neurotoxic properties, are reported to be activated through pathways such as NF-κB, express high levels of C3 and SAA3, and release inflammatory mediators including IL-1β and TNF-*α*. In contrast, A2 astrocytes are characterized as displaying anti-inflammatory and neuroprotective features, are suggested to be regulated by signaling pathways such as PI3K/AKT, express markers including S100a10 and Tgm1, and contribute to tissue repair (57). Preclinical investigations indicate that during NP progression, microglia-derived factors such as IL-1α, TNF-α, and C1q drive astrocytes toward A1 polarization (58). In animal models, A1 astrocytes exacerbate pain through several mechanisms: including cytokine-mediated sensitization of dorsal horn neurons, suppression of GLT-1 and GLAST expression leading to synaptic glutamate accumulation and excitotoxicity, and secretion of chemokines such as MCP-1 and CCL2 that recruit immune cells and amplify neuroinflammation (59, 60). Activation of P2X7 receptors has been shown to further potentiates A1 -like astrocytic responses in experimental settings (61). Notably, recent experimental evidence suggests that A1 astrocytes sustain their pro-inflammatory phenotype through a complement-mediated positive feedback loop involving the C3-C3aR axis, and pharmacological blockade of C3aR effectively alleviates pain behaviors (62). Although these mechanistic insights are compelling, confirmation of a comparable C3-C3aR-driven astrocytic loop in human NP patients is currently lacking (Figure 3) (Table 1).

Conversely, A2 astrocytes have been implicated in exerting neuroprotective effects by limiting excitotoxicity and suppressing inflammation through secretion of neurotrophic factors, including BDNF, GDNF, and nerve growth factor (NGF), upregulation of EAAT2 to enhance glutamate clearance, and release of anti-inflammatory mediators such as IL-10 and TGF-*β* in preclinical models (63, 64). Additionally, experimental studies suggest that A2 -like astrocytes support extracellular matrix remodeling and promote tissue repair. Whether these reparative functions are recapitulated in the human CNS and can be therapeutically harnessed to alleviate chronic pain awaits clinical investigation (Figure 3; Table 1).

#### 3.4.3 Dynamic regulation of astrocytic phenotypic state and the limitations of binary classification

Preclinical evidence indicates that NP progression and outcome depend on the dynamic balance between pro-inflammatory and pro-reparative astrocytic phenotypes, which is often approximated by the A1/A2 ratio. Molecules including inflammasomes such as NLRP3, lipocalin-2 (Lcn2), and prokineticin 2 (PK2) have been shown in animal studies to regulate this polarization process (65, 66). Given the constraints of the A1/A2 binary model discussed above, future work should increasingly focus on defining the specific transcriptomic and epigenomic signatures of astrocytic reactivity in human NP tissues, rather than relying solely on a limited set of canonical markers.

As an illustration of the limitations inherent in extrapolating from binary classification schemes, postmortem human brain tissue analyses in autism spectrum disorder (ASD) have revealed a significant reduction in astrocyte numbers alongside a concurrent increase in GFAP^+^ immunoreactivity—a marker of heightened activation—within prefrontal cortical regions (BA9, BA46, BA47) compared to age- and sex-matched controls (67). These observations underscore that human astrocytes in neurological disease contexts exhibit complex structural and reactive alterations that cannot be adequately captured by the simple A1/A2 dichotomy. This further emphasizes the need for more nuanced, transcriptomically informed approaches to characterize astrocytic states in human pain conditions.

#### 3.4.4 Therapeutic implications and future directions

Current therapeutic strategies therefore aim to suppress A1-like polarization—for example through preclinical P2X7 receptor or NLRP3 inhibition—or to enhance A2-like polarization by administration of neurotrophic factors in animal models, thereby restoring phenotypic balance (Table 1). While reestablishing a favorable equilibrium between reactive astrocyte states represents a promising therapeutic avenue, translation to clinical practice will necessitate a deeper, more nuanced understanding of human astrocytic heterogeneity and the development of validated biomarkers that extend beyond the A1/A2 paradigm.

### 3.5 Heterogeneity of other glial types and NP

#### 3.5.1 Oligodendroglial regional heterogeneity and homeostatic functions

**Figure 4:**
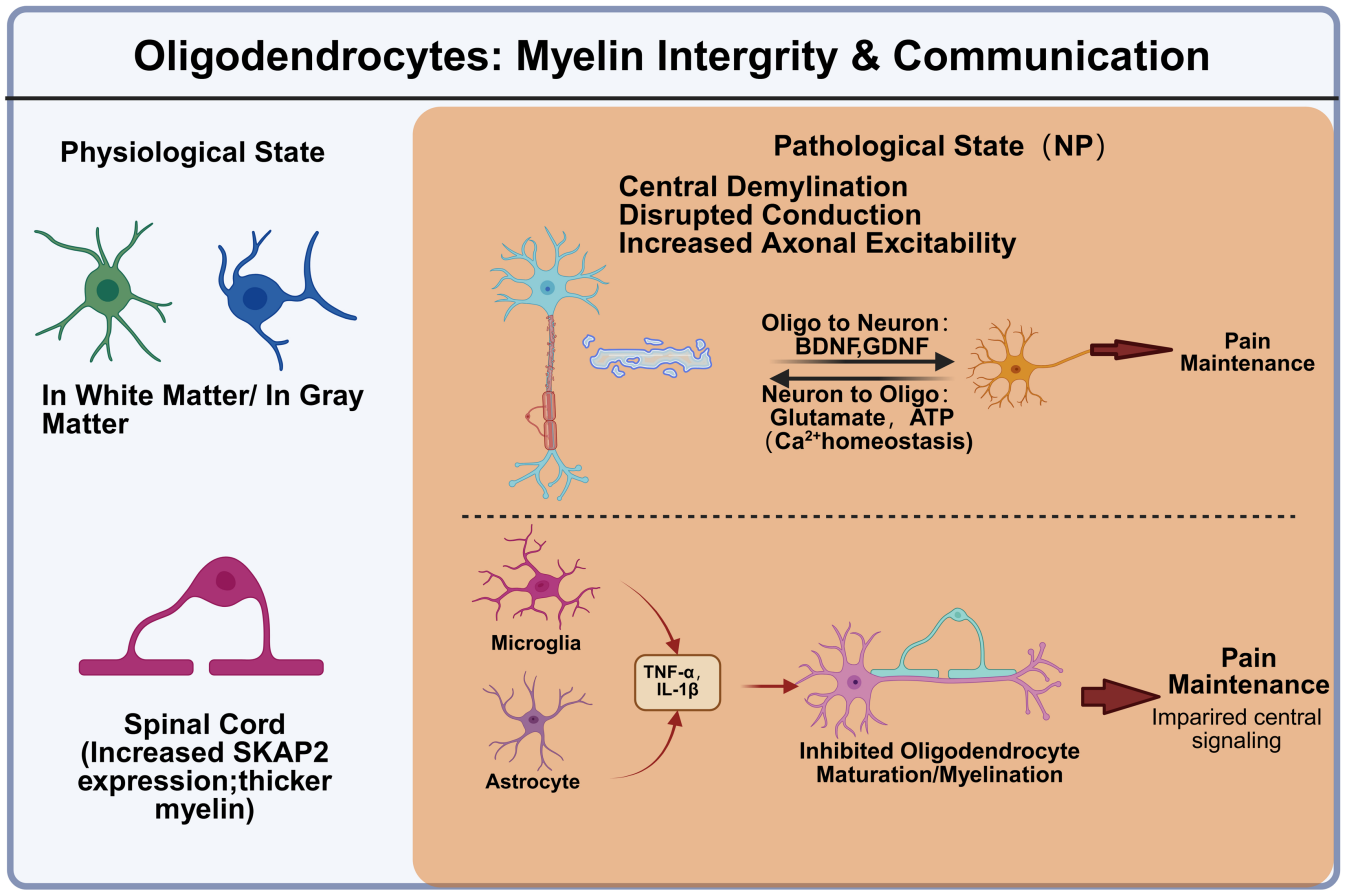
Physiological manifestations of oligodendrocyte heterogeneity and its pathological changes in NP. This figure summarizes key roles of oligodendrocyte in NP. Oligodendrocytes exhibit regional heterogeneity across gray and white matter. Spinal cord OPCs uniquely express SKAP2. In neuropathic pain, central demyelination, impaired remyelination, and pro-inflammatory cytokines (TNF-α, IL-1β) from activated glia disrupt function. Bidirectional signaling via neurotrophic factors (BDNF, GDNF) and neuronal signals (glutamate, ATP) is involved. Preclinical studies link oligodendrocyte loss and myelin thinning to pain, but human evidence remains scarce.

Oligodendrocytes are essential CNS cells responsible for myelin sheath formation, enabling rapid and efficient action potential conduction. Their abundance, differentiation dynamics, and functional properties vary markedly across brain and spinal cord regions, such as gray and white matter, reflecting intrinsic spatiotemporal heterogeneity (68–70). Recent single-nucleus RNA sequencing of human post-mortem white matter from brain, cerebellum, and spinal cord has revealed that region-specific oligodendrocyte precursor cells (OPCs) give rise to distinct oligodendrocyte populations; notably, spinal cord oligodendrocytes exhibit unique markers such as SKAP2 (71). Moreover, single-cell analyses of human cortical/subcortical and spinal cord samples have identified regional and age-related transcriptional diversity among mature oligodendrocytes (72) (Figure 4; Table 2).

**Table 2:** Heterogeneity of other glial types (oligodendrocyte lineage and satellite glial cells) and their roles in neuropathic pain.

Cell type	Polarization/activation state	Markers	Functional characteristics	References
Oligodendrocytes	Physiological regional heterogeneity	Spinal cord specific: SKAP2	Myelin sheath formation; region-specific diversity across brain, cerebellum, spinal cord, gray/white matter	(68–72)
Oligodendrocytes	NP-associated–Myelin damage and impaired remyelination	Not specified	Peripheral nerve injury induces central demyelination, disrupts sensory conduction, reduces velocity, increases axonal excitability; aberrant OPC proliferation impairs remyelination	(73)
Oligodendrocytes	NP-associated–Suppressed maturation by inflammatory mediators	Not specified	TNF-α and IL-1β from microglia/astrocytes suppress oligodendrocyte maturation and myelination capacity	(74)
Oligodendrocytes	NP-associated–Bidirectional neuron-oligodendrocyte signaling	Not specified	Oligodendrocytes release BDNF, GDNF influencing plasticity; neuronal activity (glutamate, ATP) regulates oligodendrocyte Ca^2^⁺ homeostasis and metabolism	(75, 76)
Satellite glial cells (SGCs)	Physiological subtype 1	FABP7, KIR4.1, CX43	Perisomatic sheaths	(77)
Satellite glial cells (SGCs)	Physiological subtype 2	OCT6⁺	Axon initial segment	(77)
Satellite glial cells (SGCs)	Physiological subtype 3	SCN7A⁺	Nonpeptidergic neurons	(77)
Satellite glial cells (SGCs)	Physiological subtype 4	Interferon response genes	Antiviral protection	(77)
Satellite glial cells (SGCs)	Activation (satellite gliosis)–Calcium signaling	Not specified	Intracellular Ca^2^⁺ signaling increases after nerve injury, correlates with pain behaviors	(78)
Satellite glial cells (SGCs)	Activation–Enhanced gap junction communication	Connexin 43 (Cx43) upregulation	Strengthens electrical coupling and chemical signaling between SGCs, forming functional glial network facilitating pain propagation	(79, 80)
Satellite glial cells (SGCs)	Activation–Release of pro-inflammatory mediators	GFAP (upregulated)	Activated by substance P via NK-1 receptors; secrete IL-1β, TNF-α, CCL2; modulate neuronal ion channels (TRPV1, Nav1.8); recruit immune cells	(81–83)
Satellite glial cells (SGCs)	Activation–Specific signaling axes: AOPPs/RAGE/NF-κB	Not specified	AOPPs exacerbate SGC activation and NP via RAGE/NF-κB (rodent CCI models)	(84)
Satellite glial cells (SGCs)	Activation–Specific signaling axes: NR2A-Wnt-TLR2	Not specified	Promotes peripheral sensitization in diabetic NP models	(85)
Satellite glial cells (SGCs)	Activation–Disruption of supportive functions	Not specified	Impairment of glutamate-glutamine cycle and potassium homeostasis contributes to sustained neuronal hyperexcitability	(81–83)

#### 3.5.2 Oligodendroglial dysfunction in NP: evidence from preclinical models

Under NP conditions, preclinical studies demonstrate that oligodendrocytes contribute to pathological processes through several mechanisms. First, myelin damage occurs, as peripheral nerve injury can induce central demyelination that disrupts sensory conduction pathways, reduces conduction velocity, and increases axonal excitability. Aberrant proliferation and differentiation of oligodendrocyte precursor cells further impair effective remyelination (73). Second, experimental evidence shows that pro-inflammatory mediators including TNF-*α* and IL-1β released by activated microglia and astrocytes suppress oligodendrocyte maturation and myelination capacity (74). Third, intercellular communication contributes to dysfunction, as oligodendrocytes influence neuronal plasticity through release of neurotrophic factors such as BDNF and GDNF, whereas neuronal activity-dependent signals including glutamate and ATP regulate oligodendrocyte calcium homeostasis and metabolic activity, forming bidirectional signaling interactions (75, 76). Evidence from preclinical models of peripheral nerve injury, spinal cord injury, chemotherapy, and HIV-associated neuropathy suggests that reduced oligodendrocyte numbers and diminished myelin thickness may contribute to pain development or maintenance (73). However, direct evidence linking oligodendroglial pathology to pain outcomes in human patients remains sparse (Figure 4; Table 2).

#### 3.5.3 Satellite glial cell heterogeneity and activation in NP

**Figure 5:**
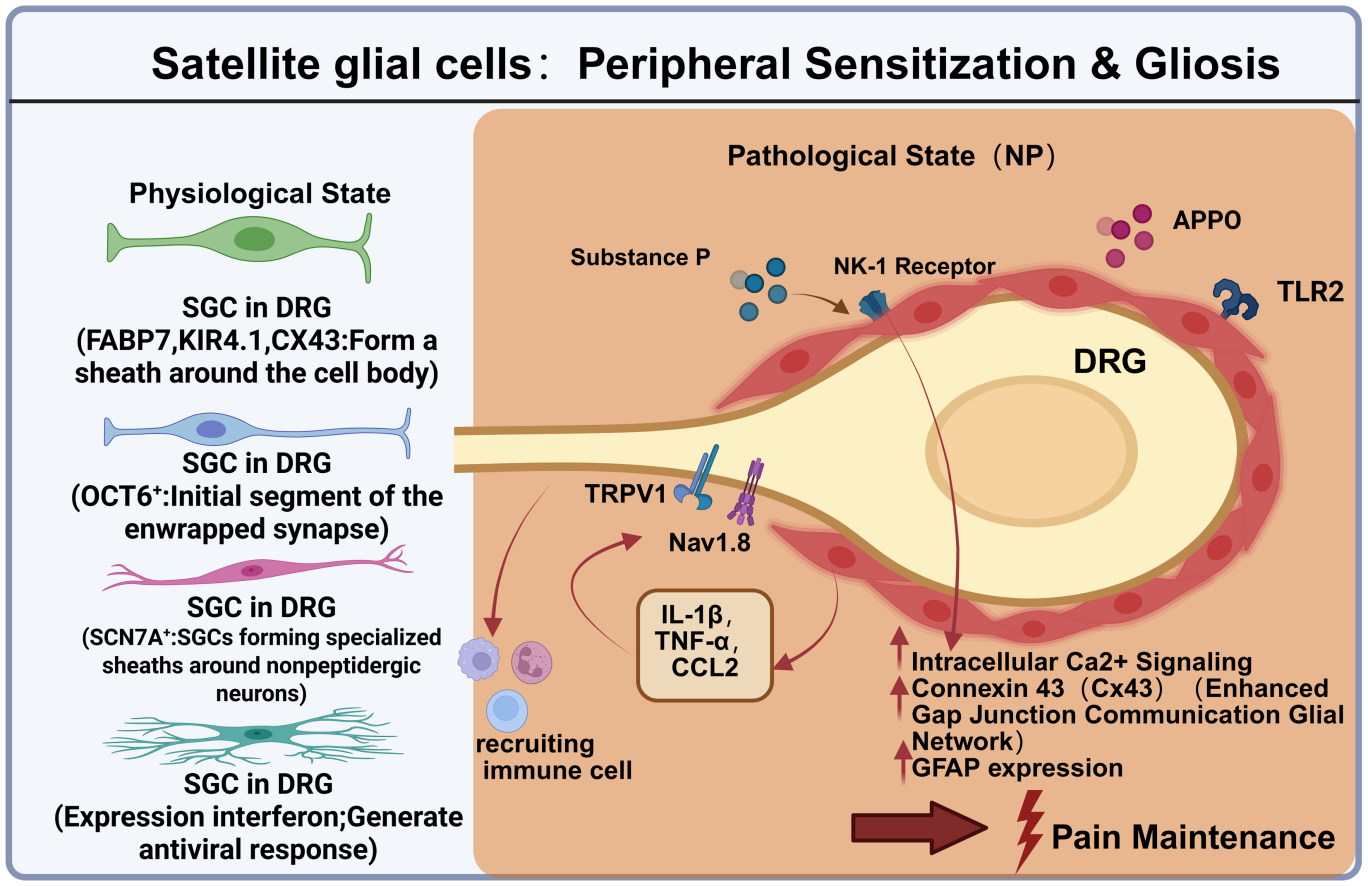
Physiological manifestations of satellite glial cell heterogeneity and its pathological changes in NP. This figure summarizes key roles of satellite glial cell in NP. Satellite glial cells (SGCs) envelop sensory neuron somata. Single-cell profiling reveals four mouse DRG SGC subtypes with distinct spatial organization: perisomatic sheaths (FABP7^+^), initial segment (OCT6^+^), SCN7A^+^, and interferon-responsive. Human DRG displays inner/outer layers. In neuropathic pain, SGCs undergo gliosis featuring elevated calcium signaling, enhanced Cx43 gap junctions, and secretion of pro-inflammatory mediators (IL-1β, TNF-α, CCL2). Signaling axes (RAGE/NF-κB, NR2A-Wnt-TLR2) drive activation, while impaired glutamate/potassium homeostasis promotes hyperexcitability. Clinical relevance awaits validation.

SGCs envelop the somata of peripheral sensory neurons within the DRG and trigeminal ganglion. A recent study integrating single-cell RNA sequencing with immunohistochemistry and advanced imaging has provided the first comprehensive *in situ* characterization of SGC heterogeneity in the mouse DRG, identifying four distinct subtypes with unique spatial organization: (i) predominant perisomatic sheaths expressing FABP7, KIR4.1, and CX43; (ii) OCT6^+^ SGCs ensheathing initial axon segments; (iii) SCN7A^+^ SGCs forming specialized sheaths around nonpeptidergic neurons, suggesting a role in itch signaling; and (iv) interferon response gene-expressing SGCs potentially involved in antiviral protection. Human DRG analyses further reveal distinct inner and outer perisomatic layers with differential marker expression (77) (Figure 5; Table 2).

In NP, preclinical evidence indicates that SGCs become activated and undergo satellite gliosis, thereby exacerbating pain through multiple mechanisms. First, intracellular calcium signaling is markedly increased following nerve injury and correlates with pain behaviors, reflecting altered metabolic activity and intercellular communication (78). Second, enhanced gap junction communication occurs, as upregulation of connexin 43 (Cx43) strengthens electrical coupling and chemical signaling between SGCs, forming a functional glial network that facilitates pain signal propagation (79, 80). Third, SGCs release pro-inflammatory mediators. Activated by neuronal signals such as substance P through NK-1 receptors and MAPK pathways, SGCs upregulate GFAP expression and secrete inflammatory mediators, including IL-1β, TNF-*α*, and CCL2. These mediators directly modulate neuronal ion channels such as TRPV1 and Nav1.8, increasing neuronal excitability and recruiting immune cells to amplify inflammation (81–83). Recent studies have elucidated specific signaling axes mediating SGC activation: advanced oxidation protein products (AOPPs) exacerbate SGC activation and NP via RAGE/NF-κB signaling in rodent CCI models (84), while the NR2A-Wnt-TLR2 signaling axis in DRG SGCs promotes peripheral sensitization in diabetic NP models (85). Impairment of SGC supportive functions, including disruption of the glutamate-glutamine cycle and potassium homeostasis, contributes to sustained neuronal hyperexcitability (Figure 5; Table 2). However, whether these subtype-specific SGC functions are conserved in human pain conditions and can be therapeutically targeted awaits clinical validation.

### 3.6 Integrated glial crosstalk in NP: coordinated cellular networks and translational considerations

The pathogenesis of NP is not driven by any single glial population in isolation but rather by coordinated, bidirectional interactions among microglia, astrocytes, oligodendrocytes, and satellite glial cells (SGCs) across both central and peripheral compartments. Within the spinal dorsal horn, activated microglia serve as the initial responders to peripheral nerve injury, releasing IL-1α, TNF, and C1q that drive astrocytes toward a pro-inflammatory phenotype, thereby amplifying and sustaining central sensitization (86). This microglia-astrocyte signaling axis is further reinforced by convergent pathways such as TLR2-mediated neuroimmune activation, wherein damage-associated molecular patterns trigger coordinated inflammatory cascades in both cell types (87). Concurrently, spinal dorsal horn neurons, microglia, and astrocytes synergize with Schwann cell-derived signals to process nociceptive information, with aberrant neuronal activity compromising myelin integrity and trophic support, thereby engaging oligodendrocytes in a pathological feedback loop (26). In the periphery, SGCs in the dorsal root ganglion communicate extensively with sensory neurons via connexin 43-mediated gap junctions, and recent evidence demonstrates that SGCs can also transfer mitochondria to these neurons through tunneling nanotubes, a process that is compromised in diabetic neuropathy.

Nevertheless, the translational relevance of these integrated glial networks remains constrained by several factors. Most mechanistic insights derive from preclinical rodent models; direct evidence for coordinated glial crosstalk in human NP tissues is sparse. Furthermore, the relative contributions of individual glial subtypes versus their collective network properties to pain chronicity remain poorly defined.

## 4 Clinical correlations of glial heterogeneity and NP

### 4.1 Clinical evidence for glial activation in human NP

Clinical evidence indicates that persistent glial activation represents a core pathological feature in NP development. Nerve injury or disease induces marked reprogramming of receptor and ion channel expression in microglia and astrocytes within the CNS (88). These molecular alterations promote glial proliferation and sustain neuroinflammatory cascades, thereby contributing to pain chronicity. Clinically, dynamic changes in glial activation markers within the spinal cord and brain can be detected through cerebrospinal fluid biomarker analysis and neuroimaging modalities such as positron emission tomography, providing objective support for NP pathophysiological assessment (89). For instance, a recent clinical study using [^11^C] DPA713 PET/CT quantified TSPO binding as a marker of microglial activation in the spinal cord and neuroforamina of patients with painful cervical radiculopathy, demonstrating significantly elevated neuroinflammation in affected tissues compared to unaffected controls (90). Microglial proliferation in the spinal dorsal horn following injury constitutes a key initiating event in pain development (33), whereas astrocytes remodel nociceptive synaptic transmission through complex neuron-glial and glial-glial communication networks, continuously amplifying pain signaling during the chronic phase (15).

### 4.2 Etiology-dependent heterogeneity of glial responses

Notably, glial response patterns exhibit pronounced heterogeneity across NP of distinct etiologies, including diabetic neuropathy and postherpetic neuralgia. Evidence indicates that glial cells are critically involved following nerve injury caused by diabetes, chemotherapy, major surgery, or viral infection; however, activation states, mediator profiles, and temporal dynamics vary according to etiology. Of particular importance, SGCs in the peripheral nervous system share certain molecular features and functional roles with central astrocytes but do not simply replicate their mechanisms (91, 92). Instead, SGCs contribute to pain through distinct mediator repertoires, compartment-specific neuronal interactions, and activation within defined post-injury time windows (80). For example, recent work has demonstrated that galactosylceramide (GalCer) depletion in SGCs is a central mediator of SGC dysfunction in painful diabetic peripheral neuropathy, disrupting neuron–glia interactions and exacerbating NP (93). This etiology- and cell type–specific heterogeneity enhances understanding of NP pathogenesis and provides a strong scientific foundation for the development of precise and individualized therapeutic strategies.

## 5 Therapeutic strategies targeting glial heterogeneity for NP

### 5.1 Limitations of current first-line therapies and the rationale for glial targeting

Current first-line therapies for NP, including gabapentin, pregabalin, and selected sodium channel blockers, primarily reduce neuronal excitability, yet their clinical efficacy is often limited by adverse effects and interindividual variability (2, 94). A fundamental limitation of these approaches is their failure to adequately address persistent glial activation and glia-driven neuroinflammation, which constitute central pathological mechanisms underlying NP chronicity and treatment resistance. Consequently, a therapeutic shift from neuron-centered suppression toward modulation of glial networks has emerged as a critical unmet need.

Advances in understanding glial heterogeneity and functional specialization have enabled development of targeted interventions aimed at specific cell types and activation states (95). In pro-inflammatory microglia, preclinical studies have shown that antagonism of purinergic receptors such as P2X4R, for example, using TNP-ATP, suppresses pathological activation and abnormal BDNF release, thereby alleviating pain and attenuating neuroinflammation. In astrocytes, inhibition of pro-inflammatory polarization represents a key strategy (96, 97). Experimental evidence indicates that targeting NF-κB signaling, for instance, with BAY-117082, markedly reduces astrocyte-mediated neuroinflammation and pain. Conversely, activation of pro-repair pathways such as PI3K/AKT, for example via IGF-1, or correction of metabolic dysregulation through restoration of PTEN expression and cholesterol homeostasis, has been shown in preclinical settings to promotes astrocytic neuroprotective functions and reestablishes cellular homeostasis within the pain microenvironment (12).

### 5.2 The translational gap: clinical failures of glial-targeted therapies

Despite the breadth of preclinical evidence supporting glial modulation as a therapeutic strategy, translation to clinical practice has proven challenging. A 2025 systematic review of 26 randomized controlled trials (2,132 participants) evaluating glial-modulating agents—including minocycline, pentoxifylline, and ibudilast—concluded that current evidence does not provide convincing support for the efficacy of pharmacological glial modulation in pain treatment or prevention (98). Among these trials, only six reported a positive treatment effect, whereas 11 yielded mixed results and 9 found no benefit compared to placebo. These disappointing outcomes highlight a persistent translational gap between promising preclinical findings and meaningful clinical efficacy.

Several factors likely contribute to this gap. Species divergence in glial biology between rodents and humans limits direct extrapolation of preclinical results, as evidenced by comparative single-nucleus transcriptomic analyses revealing substantial heterogeneity in all glial cell types between human and mouse spinal cords (13). Moreover, the spatiotemporal dynamics of glial activation in human NP remain poorly defined, obscuring optimal therapeutic windows. The absence of validated biomarkers for monitoring glial states further impedes patient stratification and assessment of target engagement. Collectively, these barriers underscore the need for more refined translational approaches that account for human-specific glial biology and etiology-dependent heterogeneity.

### 5.3 Harnessing glial heterogeneity for individualized therapy

The recognition that glial responses vary substantially across NP etiologies and among individual patients has catalyzed a shift toward precision-based therapeutic strategies. Single-cell and single-nucleus multiomic atlases of human dorsal root ganglia have recently mapped the transcriptomic and chromatin accessibility landscapes of non-neuronal cell types, including satellite glial cells, fibroblasts, and macrophages, revealing that these populations critically contribute to pain pathogenesis and represent previously underappreciated therapeutic targets (99). Such molecular atlases enable identification of patient-specific glial signatures that may predict treatment responsiveness. Profiling patient-specific glial subpopulations and transcriptional signatures enables identification of molecular drivers underlying individual pain phenotypes (19). Building on these insights, gene- or cell-based interventions targeting defined molecular functions, such as PTEN signaling, or metabolic pathways, including cholesterol balance and FXR signaling, represent promising approaches for achieving precision analgesia (100, 101). Collectively, therapeutic strategies targeting glial heterogeneity offer a path to overcome current limitations in NP management and signify a conceptual transition from symptomatic relief toward correction of underlying disease mechanisms. Achieving this goal will require the integration of human-relevant experimental models, stratification of patients with different NP subtypes, and identification of reliable common biomarkers capable of guiding glial-targeted interventions in the clinical setting.

## 6 Discussion

NP is a chronic pain syndrome arising from damage or disease of the somatosensory system and imposes a substantial clinical and socioeconomic burden due to its high prevalence and frequent refractoriness. The recognition that glial cells, including microglia, astrocytes, oligodendrocytes, and satellite glial cells, exhibit pronounced heterogeneity across cell types, anatomical regions, and functional states has significantly deepened our understanding of NP pathophysiology. Rather than constituting a passive or uniform response, glial activation in NP encompasses a continuum of context-dependent transcriptional and phenotypic states that collectively form a complex regulatory network governing pain initiation, transmission, and persistence. This heterogeneity provides critical insight into both disease mechanisms and therapeutic innovation, while also revealing important conceptual limitations of binary classification frameworks that have historically dominated the field.

This review summarizes the diverse mechanisms by which glial heterogeneity contributes to NP. Within the CNS, the dynamic balance between pro-inflammatory and pro-reparative microglial phenotypes sculpts neuroinflammatory responses, and this equilibrium is more faithfully represented by disease-associated transcriptional signatures than by rigid M1/M2 labels. Astrocytes exert bidirectional effects through neurotoxic or neuroprotective functional orientations, and emerging evidence indicates that their reactive states in human neurological conditions involve complex structural and molecular alterations that are not fully captured by the A1/A2 dichotomy. Oligodendroglial heterogeneity has recently been mapped at single-nucleus resolution across human brain and spinal cord regions, revealing region-specific vulnerabilities that may differentially impact myelin integrity and axonal conduction in distinct NP etiologies. In the peripheral nervous system, SGCs comprise molecularly distinct subtypes that include subpopulations specialized for perisomatic ensheathment, axonal initial segment support, and interferon-mediated immune surveillance. These subtypes sensitize sensory neurons through enhanced gap junction coupling, release of inflammatory mediators, and impairment of intercellular mitochondrial transfer. A particularly compelling insight emerging from recent work is the recognition that these heterogeneous glial populations do not act in isolation. Coordinated glial–glial crosstalk, exemplified by microglia-derived signals that drive astrocytic reactivity and oligodendroglial susceptibility as well as SGC–neuron metabolic coupling in the periphery, constitutes a higher-order level of regulatory complexity that remains incompletely understood. Together, these convergent mechanisms drive key pathological processes, including synaptic remodeling, neuroinflammatory cascades, and ion channel dysregulation, ultimately leading to peripheral and central sensitization and impaired descending inhibition.

Advances in deciphering glial heterogeneity are facilitating a conceptual transition in NP management from symptomatic relief toward mechanism-based intervention. Clinical evidence indicates that distinct etiologies of NP result in different glial activation profiles, suggesting that biomarker-guided personalized therapeutic strategies may be feasible. For example, differential SGC metabolic reprogramming has been documented in diabetic neuropathy compared with post-herpetic neuralgia. Emerging approaches targeting specific glial subpopulations or state transitions, including P2X4R antagonists,and interventions informed by single-cell omics profiling, show promise beyond conventional neuron-directed pharmacotherapy. Nevertheless, the translational landscape is tempered by sobering clinical realities. Multiple glial modulators with robust preclinical efficacy, including propentofylline and minocycline, have failed to demonstrate meaningful analgesic benefit in human NP trials. These setbacks underscore a persistent translational gap that may be attributable, at least in part, to species divergence in glial biology, the absence of validated biomarkers for patient stratification, and an incomplete appreciation of the spatiotemporal dynamics governing human glial reactivity.

Despite this progress, substantial challenges and knowledge gaps remain. The precise signaling networks that coordinate SGC subtype specification and function in human sensory ganglia are largely undefined. The temporal evolution and molecular choreography of glial–glial crosstalk during the transition from acute to chronic pain have yet to be systematically delineated. Furthermore, the specific contributions of oligodendroglial heterogeneity and myelin plasticity to NP chronicity represent a nascent area of investigation with considerable therapeutic implications. Future research should prioritize the integration of high-resolution spatiotemporal omics with advanced live imaging in human-relevant model systems to construct a dynamic, multi-dimensional atlas of the evolving glial landscape in NP. Equally important will be the identification of conserved, targetable nodes within glial regulatory networks that may transcend species and etiological differences. Translating these insights into clinical practice through the development of selective glial modulators, rational combination strategies, and precision patient selection will require sustained effort. Ultimately, a more comprehensive and nuanced understanding of glial heterogeneity, one that better accounts for its continuum nature and network-level complexity, may contribute to achieving precision analgesia and reducing the global burden of NP.

## Glossary

AKT: Protein Kinase B
Ang-1: Angiopoietin-1
AOPPs: Advanced Oxidation Protein Products
Arg-1: Arginase-1
ASD: Autism Spectrum Disorder
ATP: Adenosine Triphosphate
BA9/BA46/BA47: Brodmann Area 9/46/47
BDNF: Brain-Derived Neurotrophic Factor
C1q: Complement Component 1q
C3: Complement Component 3
C3aR: Complement Component 3a Receptor
Cav3.2: Voltage-Gated Calcium Channel 3.2
CCI: Chronic Constriction Injury
CCL2: C-C Motif Chemokine Ligand 2
CD86: Cluster of Differentiation 86
CD206: Cluster of Differentiation 206
CNS: Central Nervous System
CX3CL1: C-X3-C Motif Chemokine Ligand 1
CX3CR1: C-X3-C Motif Chemokine Receptor 1
CX43/Cx43: Connexin 43
DRG: Dorsal Root Ganglion
EAAT2: Excitatory Amino Acid Transporter 2
FABP7: Fatty Acid Binding Protein 7
FXR: Farnesoid X Receptor
GABA: *γ*-Aminobutyric Acid
Gal3: Galectin-3
GalCer: Galactosylceramide
GAT-1: GABA Transporter 1
GDF11: Growth Differentiation Factor 11
GDNF: Glial Cell Line-Derived Neurotrophic Factor
GFAP: Glial Fibrillary Acidic Protein
GLAST: Glutamate Aspartate Transporter
GLT-1: Glutamate Transporter 1
HIV: Human Immunodeficiency Virus
IGF-1: Insulin-Like Growth Factor 1
IL-1α: Interleukin-1 Alpha
IL-1β: Interleukin-1 Beta
IL-6: Interleukin-6
IL-10: Interleukin-10
Il6ra: Interleukin 6 Receptor Alpha
iNOS: Inducible Nitric Oxide Synthase
JAK: Janus Kinase
Kir4.1: Inward Rectifying Potassium Channel 4.1
Kv4.2: Voltage-Gated Potassium Channel 4.2
Lcn2: Lipocalin-2
MAPK: Mitogen-Activated Protein Kinase
MCP-1: Monocyte Chemoattractant Protein-1
MMP: Matrix Metalloproteinase
MOR: *μ*-Opioid Receptor
mTOR: Mechanistic Target of Rapamycin
Nav1.3: Voltage-Gated Sodium Channel 1.3
Nav1.8: Voltage-Gated Sodium Channel 1.8
NF-κB: Nuclear Factor Kappa-Light-Chain-Enhancer of Activated B Cells
NGF: Nerve Growth Factor
NK-1: Neurokinin-1 Receptor
NLRP3: NOD-Like Receptor Family Pyrin Domain Containing 3
NMDA: N-Methyl-D-Aspartate
NP: Neuropathic Pain
NR2A: NMDA Receptor Subunit 2A
OCT6: Octamer-Binding Transcription Factor 6
OPCs: Oligodendrocyte Precursor Cells
P2X4R: Purinergic Receptor P2X4
P2X7R: Purinergic Receptor P2X7
PD-L1: Programmed Death-Ligand 1
PET/CT: Positron Emission Tomography/Computed Tomography
PI3K: Phosphoinositide 3-Kinase
PK2: Prokineticin 2
PTEN: Phosphatase and Tensin Homolog
RAGE: Receptor for Advanced Glycation End Products
S100a10: S100 Calcium Binding Protein A10
SAA3: Serum Amyloid A3
SCN7A: Sodium Voltage-Gated Channel Alpha Subunit 7
SGCs: Satellite Glial Cells
SKAP2: Src Kinase-Associated Phosphoprotein 2
STAT3: Signal Transducer and Activator of Transcription 3
TGF-*β*: Transforming Growth Factor Beta
Tgm1: Transglutaminase 1
TLR2: Toll-Like Receptor 2
TNF-*α*: Tumor Necrosis Factor Alpha
TNP-ATP: 2′,3′-O-(2,4,6-Trinitrophenyl) Adenosine Triphosphate
TRPV1: Transient Receptor Potential Vanilloid 1
TSPO: Translocator Protein
Wnt: Wingless-Related Integration Site
Ym1/2: Chitinase-Like Proteins 1/2
